# Nursing Intervention "EducaTHE" to Improve Knowledge and Self-care Behaviors for Hypertensive Disorders in Pregnant Women: a Randomized Controlled Pilot Study

**DOI:** 10.17533/udea.iee.v43n1e14

**Published:** 2025-04-29

**Authors:** Iliana Milena Ulloa Sabogal, Mauricio Arias Rojas

**Affiliations:** 1 RN, M.Sc, Ph.D. Professor, School of Nursing, Universidad Pedagógica y Tecnológica de Colombia, Tunja, Colombia. Email: iliana.ulloa@uptc.edu.co. Corresponding author. https://orcid.org/0000-0003-1605-6837 Universidad Pedagógica y Tecnológica de Colombia School of Nursing Universidad Pedagógica y Tecnológica de Colombia Tunja Colombia iliana.ulloa@uptc.edu.co; 2 RN, M.Sc., Ph.D. Associate Professor, Faculty of Nursing, Universidad de Antioquia, Medellín, Colombia. Email: emauricio.arias@udea.edu.co. https://orcid.org/0000-0003-2096-1792 Universidad de Antioquia Faculty of Nursing Universidad de Antioquia Medellín Colombia emauricio.arias@udea.edu.co

**Keywords:** randomized controlled trial, nursing, hypertension, pregnancy-induced, self-care, behavior, theory of planned behavior., ensayo controlado aleatorizado, enfermería, hipertensión inducida en el embarazo, autocuidado, comportamiento, teoría del comportamiento planificado., ensaio clínico controlado aleatório, enfermagem, hipertensão induzida pela gravidez, autocuidado, comportamento, teoria do comportamento planejado.

## Abstract

**Objective.:**

This work sought to determine the feasibility, acceptability, and potential effect of the nursing intervention “Knowledge and self-care behaviors of pregnant women in the face of hypertensive disorders of pregnancy - EducaTHE”.

**Methods.:**

This study is a pilot randomized controlled trial. Sixty pregnant women who were enrolled in the antenatal care program of a health institution participated. They were selected via simple random sampling and assigned to the experimental group (*n* = 30) and control group (*n* = 30). The intervention consisted of four educational sessions once a week, while the control group received usual antenatal care. Recruitment, follow-up, and dropout rates were assessed, as well as participant satisfaction. Both groups completed the "Knowledge and Self-Care Behaviors - CoNOCiTHE" and "Determinants of Behavior" scales before and four weeks after the intervention to assess impact.

**Results.:**

The recruitment rate was 75.94%, 90% at follow-up and 10% dropouts. Overall satisfaction was 4.82 out of 5. The effect of the intervention showed a significant increase in the level of knowledge and behaviors in the experimental group compared to the control group (77 vs. 69; *p*< 0.001) and in the determinants of behavior (159 vs. 154; *p*=0.066), and a large effect size (d-Cliff = 0.7517). These changes were not significant in the control group (*p*≥0.05).

**Conclusion.:**

This study shows the feasibility and acceptability of the intervention in the maternal population, as well as the potential effect of improving knowledge and self-care behaviors in the face of hypertensive disorders of pregnancy. Therefore, it is recommended to use these types of educational interventions in maternal and perinatal nursing care.

## Resumen

**Objetivo.:**

Determinar la factibilidad, aceptabilidad y efecto potencial de la intervención de enfermería “Conocimientos y comportamientos de autocuidado de la mujer embarazada ante los trastornos hipertensivos del embarazo - EducaTHE”.

**Métodos.:**

Este es un estudio piloto controlado aleatorizado. Participaron sesenta mujeres embarazadas inscritas en el programa de atención prenatal de una institución de salud, seleccionadas por muestreo aleatorio simple y asignadas al grupo experimental (*n* = 30) y grupo control (*n* = 30). La intervención consistió en cuatro sesiones educativas una vez por semana, mientras el grupo control recibió la atención prenatal habitual. Se evaluaron las tasas de reclutamiento, seguimiento y deserción, así como la satisfacción de las participantes. Ambos grupos diligenciaron las escalas “Conocimientos y comportamientos de autocuidado - CoNOCiTHE” y “Determinantes del comportamiento” antes y cuatro semanas después de la intervención para evaluar el impacto.

**Resultados.:**

La tasa de reclutamiento fue del 75.94 %, 90 % en el seguimiento y deserción del 10 %. La satisfacción global fue de 4.82 sobre 5. El efecto de la intervención evidenció un aumento significativo en el nivel de conocimientos y comportamientos en el grupo experimental en comparación con el grupo control (69 vs. 77; *p*<0.001) y en los determinantes del comportamiento (154 vs. 159; *p*=0.066), y un tamaño de efecto grande (d-Cliff = 0.7517).

**Conclusión:**

. Este estudio evidencia la factibilidad y aceptabilidad de una intervención de enfermería en la población materna, y el efecto potencial de mejorar los conocimientos y comportamientos de autocuidado ante los trastornos hipertensivos del embarazo. Por lo que se recomienda utilizar este tipo de intervenciones educativas en el cuidado de enfermería materno perinatal.

## Resumo

**Objetivo.:**

Determinar a viabilidade, aceitabilidade e potencial efeito da intervenção de enfermagem “Conhecimento e comportamentos de autocuidado de gestantes frente aos transtornos hipertensivos da gravidez - EducaTHE”.

**Métodos.:**

Este é um estudo piloto controlado randomizado. Participaram sessenta gestantes cadastradas no programa de pré-natal de uma instituição de saúde, selecionadas por amostragem aleatória simples e distribuídas em grupo experimental (*n*=30) e grupo controle (*n*=30). A intervenção consistiu em quatro sessões educativas uma vez por semana, enquanto o grupo controle recebeu cuidado pré-natal habitual. Foram avaliadas as taxas de recrutamento, acompanhamento e abandono, bem como a satisfação dos participantes. Ambos os grupos diligenciaram as escalas “Conhecimentos e comportamentos de autocuidado - CoNOCiTHE” e “Determinantes do comportamento” antes e 4 semanas após a intervenção para avaliar o impacto.

**Resultados.:**

A taxa de recrutamento foi de 75.94%, 90% em acompanhamento e 10% de abandono. A satisfação geral foi de 4.82 em 5. O efeito da intervenção mostrou um aumento significativo no nível de conhecimento e comportamentos no grupo experimental em comparação com o grupo controle (69 vs. 77; *p*<0.001) e nos determinantes do comportamento (154 vs. 159; *p*=0.066) e um grande tamanho de efeito (d-Cliff = 0.7517).

**Conclusão.:**

Este estudo evidencia a viabilidade e aceitabilidade de uma intervenção de enfermagem na população materna, e o potencial efeito da melhoria do conhecimento e dos comportamentos de autocuidado diante dos transtornos hipertensivos da gravidez. Portanto, recomenda-se a utilização desse tipo de intervenção educativa na assistência de enfermagem materna e perinatal.

## Introduction

 Hypertensive disorders of pregnancy (HDP) are the second leading cause of maternal mortality and one of the leading causes of perinatal morbidity worldwide.^1^ Hypertensive disorders of pregnancy affect approximately 10% of all pregnancies and account for about 14% of maternal mortality globally.^1^In Latin America and the Caribbean, HDP account for 26% of maternal deaths; in Asia and Africa, these contribute to 9%, and 19.6% in Colombia.^2^Currently, it is clear that HDP is a multisystemic complication with multifactorial etiology.^3^Studies by Ouasmani *et al*.,^4^and Fadare *et al*.,^5^suggest that many of the complications associated with HDP are the result of negative attitudes and low knowledge about the disease, as well as the lack of care practices by pregnant women. In this sense, interventions aimed at preventing, detecting, or controlling risk must address a wide range of factors, such as improving knowledge, lifestyle modification, and multidisciplinary care.^3^

Health education is an essential component of women's care during the antenatal period.^6^One of the main objectives of health education is to improve pregnant women's knowledge, self-care behaviors, and health decision-making, which can lead to reducing morbidity and mortality, improving quality of life, and reducing health care costs.^7^However, lack of health education interventions during prenatal care, and specifically regarding HDP, may contribute not only to women’s inadequate understanding of the disease and possible health complications, but also to delay in seeking care and poor results in maternal-fetal well-being.^4^

In this sense, research^( 8-10)^ has shown positive effects of interventions and educational programs in improving the knowledge, attitudes, and practices of self-care in women with or without HDP diagnosis. However, few studies in this field have validated the intervention,^11^its measurement instruments,^12^^-^^14^ and educational materials,^13^^,^^14^ or even the use of a theoretical reference.^15^^-^^17^In this regard, Sidani and Braden^18^ have described that validating an intervention allows verifying the fidelity of its application to correctly interpret its results and for it to be transferred and reproduced in practice. Similarly, availability of instruments with proven validity and reliability enables a systematic, objective, and reliable assessment of health needs, guiding the adoption of strategies to meet said needs.^19^ Furthermore, validation of health materials seeks to guarantee a material that is in consistent with the characteristics and needs of the population, and the objectives proposed in educational interventions.^20^ Lastly, evidence has also described that interventions should be designed based on health education models and theories,^17^ given that these can influence significantly the effectiveness of health education programs.^21^For example, studies aimed at strengthening self-care behaviors during the prenatal period have used the Theory of Planned Behavior (TPB),^22^ a theoretical reference widely described in designing and evaluating educational interventions to achieve behavioral goals focused on improving the consumption of iron supplementation,^23^ oral health,^24^ promotion of breastfeeding,^25^ prevention of urinary tract infections,^26^ and self-care in women with HDP,^17^and gestational diabetes.^27^ These studies suggest that a woman's motivation to engage in self-care behaviors during the antenatal period increases with her positive attitudes toward the behavior, her social and family support, and her awareness and control of her own resources, skills, and abilities.^17^

The aforementioned research shows the development of TPB and reveals how it has been one of the most applicable cognitive-social frameworks to study human action and predict and understand health behaviors, including self-care.^27^According to the review of the published literature, within the Colombian context, no studies were found that sought to prevent or control the risk of HDP in healthy pregnant women, nor were there any interventions that integrate a theoretical basis in their design and evaluation. Thus, a need arises to design an educational intervention that increases levels of knowledge in pregnant women and promotes self-care behaviors in the face of HDP. Therefore, the aim of this study was to determine the feasibility, acceptability, and potential effect of the nursing intervention “EducaTHE” to improve knowledge and self-care behaviors against hypertensive disorders in pregnant women.

## Methods

This was a randomized controlled pilot trial study registered in ClinicalTrials.gov (NCT05837962). This study was conducted in 2023 during three months in a health institution in Piedecuesta, Santander, Colombia. This study included pregnant women aged ≥ 18 years, enrolled in the prenatal control program, gestational age ≤ 24 weeks - as determined by the date of the last menstrual period or obstetric ultrasound, and living in the urban area. The study excluded pregnant women with cardiovascular, renal, or diabetes mellitus diseases; history or diagnosis at enrollment or during the HDP study, participation in other studies or educational programs with similar topics, and score < 24 on the Mini-Mental State Test.

Drawing from studies with similar objectives,^11^^,^^15^ the sample size was estimated at 30 participants per group, resulting in a total of 60 participants.. This choice was based to the fact that feasibility and acceptability studies do not require a formal calculation of the sample size.^28^Participants were randomly assigned to the control or experimental group in a 1:1 ratio. The Randomized Control Trial mobile application was used for group assignment. Only participants in the experimental group were contacted by the interventional nurse. The research assistant and the statistician responsible for data analysis were blinded to group allocation.

### Data Collection Instruments

The instruments used in this study were the socio-demographic and clinical characteristics questionnaire, the feasibility format, the acceptability questionnaire, and the scales "Knowledge and self-care behaviors in the face of HDP-CoNOCiTHE" and "Determinants of self-care behaviors in the face of HDP". The demographic and clinical information questionnaire included variables, such as: age, nationality, marital status, education, employment status, and income. In addition, data were collected on obstetric history, gestational age, number of antenatal visits, and attendance to the antenatal care course. The feasibility of the study included recruitment, follow-up, and dropout rates of the participants, as well as fidelity of intervention delivery. 

Acceptability was assessed using a questionnaire designed by the authors. The questionnaire assessed participants' acceptability and satisfaction in the dimensions of content, activities, delivery, and advantages and disadvantages of participating in the intervention. The questionnaire consisted of 25 items, with a 5-point Likert-type response scale, where 1 means "not at all useful or strongly disagree" and 5 means "extremely useful or strongly agreed". Higher scores correspond to a higher levels of acceptability and satisfaction with the intervention. In addition, it included three open-ended questions that assessed the positive aspects of the intervention, educational materials and other information that should be included in the intervention. The questionnaire was administered upon completing the fourth educational session.

The scale "Knowledge and self-care behaviors in the face of HDP-CoNOCiTHE", was developed by Ulloa *et al*.^29^ This instrument has two dimensions: (1) knowledge in the face of HDP (30 items); (2) self-care behaviors (28 items). The first dimension has true/false response options, with correct responses scored 1 and incorrect responses scored 0, with a minimum of 0 points and a maximum of 30 points. The second dimension has yes or no response options, with scores of 2 points if it is a behavior aimed at preventing or controlling the risk of HDP or 0 if it is not, with a minimum of 0 points and a maximum of 56 points. Each dimension was scored separately and then transformed into an overall score ranging from 0 to 86; higher scores indicate higher levels of knowledge and self-care behaviors. Ulloa *et al*.,^30^ evaluated the reliability of the scale, demonstrating a Cronbach’s alpha of 0.67 and test-retest stability with an intraclass correlation coefficient (ICC) of 0.96, which reflected the scale’s acceptable reliability. 

The scale "Determinants of self-care behavior in the face of HDP" was developed by Ulloa *et al*.^31^ The instrument contains 33 items distributed into four dimensions: Attitude (7 items); Subjective Norms (12 items); Perceived Behavioral Control (6 items), and behavioral intention (8 items). Response options vary on a 5-point Likert scale (1 = very unimportant to 5 = very important / 1 = strongly disagree to 5 = strongly agree). Scores range from 33 to 165, with higher scores indicating greater intention of self-care behaviors. Ulloa *et al*.,^31^ assessed the feasibility and reliability of the scale with an overall instrument content validity ratio (CVR') of 0.90. Cronbach’s alpha was 0.80 and the test-retest stability showed an ICC of 0.99.

### Intervention

Before starting the study, participants in both groups completed the socio-demographic and clinical information questionnaire, and the scales "Knowledge and self-care behaviors in the face of HDP-CoNOCiTHE" and "Determinants of self-care behaviors in the face of HDP". The participants in the experimental group received the intervention “Knowledge and self-care behaviors in the face of hypertensive disorders of pregnancy- EducaTHE”, an intervention guided by educational-, behavioral- and motivational-type TPB. The objective of the intervention was to improve the knowledge and self-care behaviors of pregnant women in the face of HDP. 

EducaTHE was delivered by a nurse and organized into four 90-minute educational sessions, once a week for four weeks. Table 1 shows the four educational sessions and the contents that made up the EducaTHE intervention. Each session was developed during three moments; the first introduced the topic, followed by development of contents and activities, and the third included the session’s feedback and explanation of commitments. The sessions were developed into six groups of five participants and in the health institution where the participants received antenatal care. As educational material, the participants received the booklet “I decide to take care of the high pressure during pregnancy”. The booklet included five learning units: 1) knowledge about the disease; 2) behavioral control, positive attitude, and support from the partner, relatives, and friends; 3) dietary recommendations; 4) recommendations to improve sleep, rest, and physical exercise; and 5) promotion of mental health. Each unit incorporated reinforcement activities with illustrations, phrases, selection games and content relationships. Details of the booklet’s construction and validation have been described. ^(^^32^After completing the educational intervention, participants from both groups were contacted to complete the post-test questionnaires. The experimental group was contacted four weeks after the end of the intervention, and the control group was contacted eight weeks after entering the study. The control group did not participate in the educational intervention, but continued to receive the usual care and education provided in the antenatal care and maternity and paternity preparation course, as did the experimental group.

**Table 1:** Description of the educational sessions of the EducaTHE intervention

Session	Content	Educational strategies
Session 1. Educational support		
Knowing about hypertensive disorders of pregnancy	• Etiology of the HDP • Risk Factors • Signs and symptoms • Maternal and fetal complications	Educational booklet: "I decide to take care of the high pressure during pregnancy" Video: Seven symptoms that every pregnant woman should know PowerPoint presentation
Session 2: Behavioral and motivational support		
Control over behavior, positive attitude, and support from partner, family, and friends	• Control over behavior • Attitude toward behavior •Social and family support for behavior • Intention toward behavior	Educational booklet: "I decide to take care of the high pressure during pregnancy" Video: Throw yourself PowerPoint presentation
Session 3: Educational, behavioral, and motivational support		
**Dietary recommendations, sleep, rest and exercise**	Dietary recommendations • Food groups according to the “Healthy Colombian family dish” • Dietary recommendations to prevent or control of the risk of HDP • Food preparation and consumption recommendations Recommendations for sleep, rest and exercise • Importance and benefits of sleep, rest, and exercise during pregnancy • Recommendations for maintaining good sleep and rest patterns • Physical activity: exercise routine during pregnancy	Educational booklet: "I decide to take care of the high pressure during pregnancy” Video: Healthy Colombian family dish. How to sleep during pregnancy? Exercises for pregnant women. PowerPoint presentation
Session 4: Educational, behavioral and motivational support		
Mental health promotion	• Defining stress, anxiety, and depression during pregnancy •Strategies for managing stress, anxiety, and depression • Practical activity: Breathing, relaxation, and meditation exercises	Educational booklet: " I decide to take care of the high pressure during pregnancy" Video: Stress and anxiety: What is it and what can we do? Depression during pregnancy. Guided relaxation during pregnancy. Music for pregnant women and newborns. Eight healthy habits to improve your mental health. PowerPoint presentation

### Statistical Analysis

To assess feasibility and acceptability, descriptive statistics were used to summarize sample characteristics, eligibility, recruitment, follow-up, dropout, and acceptability rates. The responses to the open-ended questions were organized according to their frequency of occurrence. 

To determine the potential effect of the intervention, the data were analyzed according to the intention-to-treat principle. The (2 test or Fisher's exact test was used to compare baseline characteristics between the groups. Normality of the data was assessed with the Kolmogorov-Smirnov test, which indicated that the data did not fit the normal model. The Mann-Whitney U test was used to determine changes between the groups. The Wilcoxon signed rank test was used for intra-group comparisons. Estimation of the effect size of the intervention on the study variables was determined using Cliff's Delta statistic (D-cliff).^33^ Data were statistically analyzed using SPSS software version 25. All tests were two-sided and *p*-values < 0.05 were considered statistically significant.

Ethical considerations. The study protocol was approved by the Research Ethics Committee of the Faculty of Nursing at Universidad de Antioquia (Act No CEI-FE 2021-31). Participants were informed about the study and signed the informed consent form. They were also assured of the privacy and confidentiality of the information.

## Results

In all, 54 pregnant women completed the study, 27 in the experimental group and 27 in the control group. At baseline, no significant differences existed between the groups in any of the sociodemographic or clinical characteristics. Participant information is detailed in Table 2.

**Table 2:** Demographic and clinical characteristics of participants in the experimental and control groups

Variables			**Experimental group (*n* = 27) n (%)**		**Control group (*n* = 27)** **n (%)**	***p*-value** **(2**
Mother's Age, Median (RIQ)		22 (17)	25 (16)	0.585^a^
Nationality	Colombian	23 (85.18)	22 (81.48)	0.5
	Venezuelan	4 (14.81)	5 (18.51)	
Educational level	Primary	0 (0)	1(3.7)	0.38
	Secondary	20 (74.07)	21 (77.77)	
	Higher education	7 (25.92)	5 (18.51)	
Occupation	Housewife	22 (81.48)	17(62.96)	0.102
	Student	3 (11.11)	1 (3.7)	
	Independent	1 (3.7)	6 (22.22)	
	Employee	1 (3.7)	3 (11.11)	
Civil status	Single	1 (3.7)	6 (22.22)	0.124
	Common law	23 (85.18)	18 (66.66)	
	Married	3 (11.11)	3 (11.11)	
Economic income	< 290 US$	8 (29.62)	7 (25.92)	0.238
	290 US$	13 (48.14)	18 (66.66)	
	> 290 US$	6 (22.22)	2 (7.4)	
Gestational Age (weeks), Mean (SD)		17.15 (5.04)	14.85 (4.02)	0.069^b^
Obstetric history	Primigravid	14 (51.85)	12 (55.55)	0.336
	Multigravida	13 (48.14)	15 (62.96)	
Number of antenatal visits	1-2	13 (48.14)	16 (59.25)	0.691
	≥ 3	14 (51.85)	11 (40.74)	
Maternity course	Yes	1 (3.7)	2 (7.40)	0.5
No	26 (96.29)	25 (92.59)

a Mann-Whitney U test, ^b^ Student *t* test

**Figure 1:**
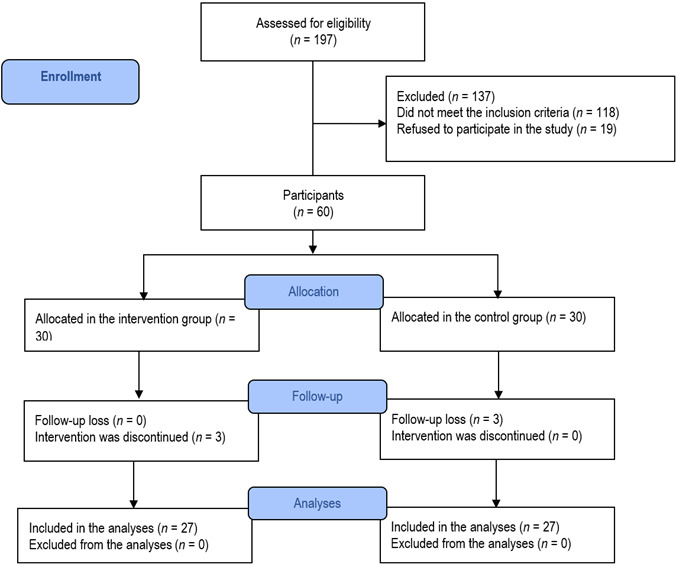
Flowchart of selection and retention of participants in the study

The trial’s feasibility is reflected by the rates achieved during its development. During the four months of the study, the recruitment rate was 75.94%. The study’s follow-up rate was 90%, with 10% dropout rate. The reasons for exclusion, refusal to participate, or failure to complete the study are described in Figure 1. Therefore, 54 participants (27 in the experimental group and 27 in the control group) completed the educational intervention protocol and were included in the analyses. The number of sessions, frequency, and estimated time to conduct the intervention were carried out according to the intervention’s protocol.

According to the acceptability, 27 participants from the experimental group answered the questionnaire. The overall acceptability of the intervention was 4.82 points out of 5. The evaluation of acceptability in all its components showed a high level of acceptance of the intervention by the participants, as detailed in Table 3. The following comments are for each of the open-ended questions. 1) What did you like most about the intervention? The answers included clear and simple explanations and recommendations (*n* = 5); dynamics, activities, and games (*n* = 5); sharing with other pregnant women (*n* = 4); the nurse's empathy, knowledge, and kindness (*n* = 4); and the opportunity to ask questions and be listened to (*n* = 1). 2) What did you like the most about the content of the booklet? Their answers detailed: the content on HDP (*n* = 8); nutritional care (*n* = 7); exercise (*n* = 6); and sleep (*n* = 2); activities of review, organization, and ease of understanding the topics (n = 4). 3) What would you have liked to have done differently? Among the answers that stand out: a greater number of sessions of the intervention and that they were spread throughout the pregnancy (*n* = 5). In addition to this question, participants expressed satisfaction with the information received, the pleasure of participating, and the positive impact on their knowledge and care actions in the face of HDP (*n* = 11). In addition, they would be willing to participate in a similar study in the future (*n* = 6).

**Table 3:** Participants' acceptance with the "EducaTHE" intervention

Dimension	Number of items	Minimum value	Maximum value	Mean (SD)
1. Contents of the intervention	7	30	35	33.70 (1.35)
2. Activities in the development of the contents	3	12	15	14.25 (0.71)
3. Delivery method	3	12	15	14.25 (0.81)
4. Advantages of the intervention	7	29	35	33.66(1.61)
5. Disadvantages of participating in the intervention	4	13	20	19.44 (1.55)
6. Overall satisfaction	1	4	5	4.96 (0.19)

The impact of the “EducaTHE” intervention on self-care knowledge and behaviors, as well as on the determinants of behavior in the face of HDP, was analyzed through inter-group and intra-group comparisons. The results of these comparisons, both for the entire questionnaire and for its subscales, are presented in Table 4. In the post-intervention measurement, the experimental group showed a significant increase in the level of knowledge and self-care behaviors compared to the control group (77 vs.69; *p* < 0.0001). In the behavioral determinants, the intervention did not show significant effects on the total score (*p* = 0.66). 

Only the intention dimension demonstrated a significant difference between groups (38 vs. 35, *p* = 0.021). The results of the comparisons within each of the groups, before and after the intervention, revealed that the participants in the experimental group showed a significant increase in the level of knowledge and self-care behaviors in the face of HDP (*p* < 0.0001) and in the total score of determinants of behavioral intention (*p* < 0.0001) after the intervention. In contrast, in the control group, no significant differences were found in any of the variables before and after the intervention.

**Table 4:** Comparison of median scores of knowledge, behavior, and determinants of behavior in the face of HDP between the experimental and control groups before and after the intervention

Dimension	Pre-test		**Intergroup comparison Value *p*** ^*a*^	Post-test		Intergroup comparison Value *p* ^*a*^	Comparison EG* Pre-test Vs. Post-test Value *p* ^*b*^	Comparison CG* Pre-test Vs. Post-test Value *p* ^b^
	**EG (*n*=27)** **Median (IQR)**	**CG (*n*=27)** **Median (IQR)**		EG* (*n*=27) **Median (IQR)**	CG* (*n*=27) **Median (IQR)**			
Total Knowledge and self-care behaviors (0-86)	68 (25)	69 (21)	0.282	77 (14)	69 (23)	< 0.0001	< 0.0001	0.618
Knowledge Dimension (0-30)	25 (13)	27 (10)	0.219	29 (5)	26 (9)	< 0.0001	< 0.0001	0.741
Behavior dimension (0-56)	42 (20)	44 (18)	0.438	48 (12)	44 (20)	< 0.001	< 0.0001	0.847
Total Determinants of Behavior (33-165)	154 (32)	154 (28)	0.965	159 (16)	154 (46)	0.066	< 0.0001	0.129
Attitude dimension (7-35)	33 (7)	34 (9)	0.294	35 (3)	34 (17)	0.335	0.010	0.740
Subjective norms dimension (12-60)	57 (13)	58 (14)	0.412	58 (6)	59 (18)	0.446	0.020	0.110
Perceived behavioral control dimension (6-30)	30 (6)	30 (6)	0.465	30 (3)	30 (7)	0.423	1.000	0.704
Behavioral intention dimension (8-40)	34 (19)	34 (16)	0.503	38 (7)	35 (15)	0.021	<0.0001	0.136

a Mann-Whitney U test ^b^ Wilcoxon signed-rank test

EG*: Experimental group, GC*: Control group, IQR: Interquartile

Finally, the size of the intervention’s effect at the intergroup level displayed a large effect size in knowledge, and a large effect size in self-care behaviors with a D-Cliff of 0.7517 (*p =* 0.001). However, the intervention had no effect on the determinants of behavior with a D-Cliff = 0.290 (*p* = 0.067).

## Discussion

This pilot study was designed to evaluate the feasibility, acceptability, and potential effect of the "EducaTHE" nursing intervention based on the Planned Behavior Theory, and was aimed at improving the knowledge and self-care behaviors of pregnant women in the face of HDP. The feasibility of this study was determined by the recruitment, follow-up, and dropout rates of the participants.

The recruitment rate of this study was 75.94%, a measure considered acceptable compared to the study by Alnuaimi *et al.,* ^(^^13^where it was only 42%. High follow-up rates of 90% and low dropout rate of 10% in this study is similar to those reported by Gingras-Charland *et al*.,^14^and the study by Alnuaimi *et al*.,^13^with follow-up rates of 91.82% and 89.68%, respectively. However, this study achieved better results when compared with other studies reporting follow-up rates of 75%.^10^^,^^34^One reason that justifies the results obtained in terms of feasibility is the eight-week interval between pre- and post-intervention measurements, which can be seen as a short period that contributed to the participants completing the study. Second, having low-risk pregnancies as inclusion criteria prevented the ocurrence of complications, hospitalizations, or pregnancy loss, situations that condition the lack of continuity in the study. Third, the fact that they received the intervention in the institution where they receive prenatal care and lived near the hospital facilitated their access, did not generate transportation costs, and facilitated their participation. However, when the dropout reasons are analyzed, similarities are found with the reasons given by authors Strassberg *et al.,*^35^and Uğurlu *et al*.,^10^specifically, in the change of city, loss of contact, and lack of interest or desire to continue.

Another variable analyzed in this study is the intervention’s acceptability. The results obtained indicate that the "EducaTHE" intervention was highly acceptable among the pregnant women who received it. This finding is quite favorable considering that Mutlaq *et al*.,^7^state that although educational interventions are essential to increase women's self-knowledge and preventive behavior in the face of pregnancy-related complications, it is necessary to continue projecting studies that determine the relevance and pertinence of these interventions in the people under study, and in the generation of evidence that contributes to improving educational processes in this population. Specifically, the results of this study showed that the contents, activities, and recommendations given during the intervention were useful and easy to comprehend and implement in the practice of prenatal care; the form of delivery was appropriate and convenient, participation brought multiple benefits, and did not generate any burden in personal or family life. These aspects indicated a level of overall satisfaction with the intervention, which received an average score of 4.96 out of 5 points.

Additionally, this study investigated the potential effect of EducaTHE intervention on improving knowledge and self-care behaviors in the face of HDP, and on the determinants that influence the intention of the behavior of pregnant women. The results showed a significant change in the total score of the knowledge scale and self-care behaviors in the face of HDP in the experimental group after receiving the intervention. These findings are consistent with several studies ^3^^,^^8^^-^^10^^,^^13^^,^^15^ that report significant differences in the level of knowledge and self-care behaviors between the intervention and control groups after receiving an educational program on HDP. The finding of significant changes in this study may be related to several factors. First, the content of the intervention was aimed at describing the characteristics and practices of self-care in the face of preventing or controlling the risk of HDP. Second, the educational booklet contained easy-to-understand topics, practical activities, review exercises, explanatory drawings, motivational phrases, and a prenatal care diary. These factors, along with the opportunity for participants to ask questions and the nurse's simple and illustrative explanations may have positively contributed to the acquisition and development of knowledge and self-care behaviors in the face of HDP.

Among determinants of self-care behavior in the face of HDP, the intervention did not show significant effects on the total score or on the dimensions of attitude, subjective norms, and perceived behavioral control between both groups; only a significant difference was found in the intention dimension for the experimental group. When analyzing these results, several aspects can be highlighted. In the first of these, the experimental group showed an increased median of the total score of the behavioral determinants and its reduction in the standard deviation, an aspect not evident in the control group. This could indicate that, given that this is a pilot study and the exact sample size was not determined to detect significant differences, a larger sample size might reveal a significant difference. Another hypothesis is that these types of variables are complex to modify, because of the variety of individual and contextual factors involved and that must be considered when predicting the performance of a behavior.^36^ Moreover, in this study, the measurement of these variables was carried out one month after the intervention, which may be a short period of time in which a possible improvement could not be reflected, as has been shown in other studies measuring the effect between six weeks^25^ and up to six months after the intervention.

The practical implications of this study raise the need for nursing interventions that integrate educational, behavioral, and motivational components. First, from the educational approach, the knowledge and self-care behaviors of pregnant women in the face of HDP are strengthened. Second, at the motivational level, greater social and family support is promoted, as well as the interest of pregnant women in achieving goals and objectives related to the development of self-care behaviors. Third, in the behavioral component, pregnant women can evaluate their behavior and identify the resources, strengths, and weaknesses that facilitate or prevent them from engaging in healthy behaviors.

Among the limitations, it should be noted that this is a pilot study, therefore, the limitations are related with the development phase and its design. First, although the sample size was adequate for a pilot study, it could be considered relatively small to obtain significant results in any of the variables raised in this study. Second, the intervention included support from a family member or significant other; however, job commitments, caring for other children, or the lack of interest did not allow for this support for most of the participants. Thirdly, this study did not follow up on the variables covered over a longer period of time, which could provide new results to support decision-making in future studies. Finally, future studies should consider the results of this research to propose a controlled clinical trial to measure the effectiveness of the "EducaTHE" nursing intervention.

Conclusion. This study assessed the feasibility, acceptability, and potential impact of "EducaTHE" nursing intervention. Feasibility assessed by recruitment, follow-up, and dropout rates demonstrated the interest of pregnant women in participating in studies that support antenatal care. High levels of acceptability and satisfaction are also highlighted, indicating that this intervention is acceptable for the target population and feasible in future studies. In terms of scope, the intervention demonstrated significant improvement in the knowledge and self-care behaviors in relation to the experimental group. Nevertheless, no significant differences were found in the determinants of behavior. Based on the results herein, the authors confirm that the "EducaTHE" intervention could be taken to the next stage to evaluate its effectiveness in a larger-scale clinical trial.
